# Supplementary material for: Iterative improvement in the automatic modular design of robot swarms
Source: PeerJ Comput Sci. 2020 Dec 7;6:e322. doi: 10.7717/peerj-cs.322 (PMC7924708; doi:10.7717/peerj-cs.322)
Supplement: Supplemental Information 3 [file peerj-cs-06-322-s003.zip › argos3/doc/api/standalone/a00414_source.html]

ARGoS: plugins/robots/e-puck/simulator/epuck\_entity.cpp Source File


- Main Page
- Related Pages
- Namespaces
- Classes
- Files

- File List
- File Members

# plugins/robots/e-puck/simulator/epuck\_entity.cpp

Go to the documentation of this file.

```
00001 
00007 #include "epuck_entity.h"
00008 
00009 #include <argos3/core/utility/math/matrix/rotationmatrix3.h>
00010 #include <argos3/core/simulator/space/space.h>
00011 #include <argos3/core/simulator/entity/controllable_entity.h>
00012 #include <argos3/core/simulator/entity/embodied_entity.h>
00013 #include <argos3/plugins/simulator/entities/rab_equipped_entity.h>
00014 #include <argos3/plugins/simulator/entities/ground_sensor_equipped_entity.h>
00015 #include <argos3/plugins/simulator/entities/led_equipped_entity.h>
00016 #include <argos3/plugins/simulator/entities/light_sensor_equipped_entity.h>
00017 #include <argos3/plugins/simulator/entities/proximity_sensor_equipped_entity.h>
00018 
00019 namespace argos {
00020 
00021    /****************************************/
00022    /****************************************/
00023 
00024    static const Real BODY_RADIUS                = 0.035f;
00025    static const Real BODY_HEIGHT                = 0.086f;
00026 
00027    static const Real INTERWHEEL_DISTANCE        = 0.053f;
00028    static const Real HALF_INTERWHEEL_DISTANCE   = INTERWHEEL_DISTANCE * 0.5f;
00029    static const Real WHEEL_RADIUS               = 0.0205f;
00030 
00031    static const Real PROXIMITY_SENSOR_RING_ELEVATION       = 0.06f;
00032    static const Real PROXIMITY_SENSOR_RING_RADIUS          = BODY_RADIUS;
00033    static const CRadians PROXIMITY_SENSOR_RING_START_ANGLE = CRadians((2 * ARGOS_PI / 8.0f) * 0.5f);
00034    static const Real PROXIMITY_SENSOR_RING_RANGE           = 0.1f;
00035 
00036    static const CRadians LED_RING_START_ANGLE   = CRadians((ARGOS_PI / 8.0f) * 0.5f);
00037    static const Real LED_RING_RADIUS            = BODY_RADIUS + 0.007;
00038    static const Real LED_RING_ELEVATION         = 0.086f;
00039    static const Real RAB_ELEVATION              = LED_RING_ELEVATION;
00040 
00041    /****************************************/
00042    /****************************************/
00043 
00044    CEPuckEntity::CEPuckEntity() :
00045       CComposableEntity(NULL),
00046       m_pcControllableEntity(NULL),
00047       m_pcEmbodiedEntity(NULL),
00048       m_pcGroundSensorEquippedEntity(NULL),
00049       m_pcLEDEquippedEntity(NULL),
00050       m_pcLightSensorEquippedEntity(NULL),
00051       m_pcProximitySensorEquippedEntity(NULL),
00052       m_pcRABEquippedEntity(NULL),
00053       m_pcWheeledEntity(NULL) {
00054    }
00055 
00056    /****************************************/
00057    /****************************************/
00058 
00059    CEPuckEntity::CEPuckEntity(const std::string& str_id,
00060                               const std::string& str_controller_id,
00061                               const CVector3& c_position,
00062                               const CQuaternion& c_orientation,
00063                               Real f_rab_range,
00064                               size_t un_rab_data_size) :
00065       CComposableEntity(NULL, str_id),
00066       m_pcControllableEntity(NULL),
00067       m_pcEmbodiedEntity(NULL),
00068       m_pcGroundSensorEquippedEntity(NULL),
00069       m_pcLEDEquippedEntity(NULL),
00070       m_pcLightSensorEquippedEntity(NULL),
00071       m_pcProximitySensorEquippedEntity(NULL),
00072       m_pcRABEquippedEntity(NULL),
00073       m_pcWheeledEntity(NULL) {
00074       try {
00075          /*
00076           * Create and init components
00077           */
00078          /* Embodied entity */
00079          m_pcEmbodiedEntity = new CEmbodiedEntity(this, "body_0", c_position, c_orientation);
00080          AddComponent(*m_pcEmbodiedEntity);
00081          /* Wheeled entity and wheel positions (left, right) */
00082          m_pcWheeledEntity = new CWheeledEntity(this, "wheels_0", 2);
00083          AddComponent(*m_pcWheeledEntity);
00084          m_pcWheeledEntity->SetWheel(0, CVector3(0.0f,  HALF_INTERWHEEL_DISTANCE, 0.0f), WHEEL_RADIUS);
00085          m_pcWheeledEntity->SetWheel(1, CVector3(0.0f, -HALF_INTERWHEEL_DISTANCE, 0.0f), WHEEL_RADIUS);
00086          /* LED equipped entity */
00087          m_pcLEDEquippedEntity = new CLEDEquippedEntity(this, "leds_0");
00088          AddComponent(*m_pcLEDEquippedEntity);
00089          m_pcLEDEquippedEntity->AddLEDRing(
00090             CVector3(0.0f, 0.0f, LED_RING_ELEVATION),
00091             LED_RING_RADIUS,
00092             LED_RING_START_ANGLE,
00093             8,
00094             m_pcEmbodiedEntity->GetOriginAnchor());
00095          /* Proximity sensor equipped entity */
00096          m_pcProximitySensorEquippedEntity =
00097             new CProximitySensorEquippedEntity(this,
00098                                                "proximity_0");
00099          AddComponent(*m_pcProximitySensorEquippedEntity);
00100          m_pcProximitySensorEquippedEntity->AddSensorRing(
00101             CVector3(0.0f, 0.0f, PROXIMITY_SENSOR_RING_ELEVATION),
00102             PROXIMITY_SENSOR_RING_RADIUS,
00103             PROXIMITY_SENSOR_RING_START_ANGLE,
00104             PROXIMITY_SENSOR_RING_RANGE,
00105             8,
00106             m_pcEmbodiedEntity->GetOriginAnchor());
00107          /* Light sensor equipped entity */
00108          m_pcLightSensorEquippedEntity =
00109             new CLightSensorEquippedEntity(this,
00110                                            "light_0");
00111          AddComponent(*m_pcLightSensorEquippedEntity);
00112          m_pcLightSensorEquippedEntity->AddSensorRing(
00113             CVector3(0.0f, 0.0f, PROXIMITY_SENSOR_RING_ELEVATION),
00114             PROXIMITY_SENSOR_RING_RADIUS,
00115             PROXIMITY_SENSOR_RING_START_ANGLE,
00116             PROXIMITY_SENSOR_RING_RANGE,
00117             8,
00118             m_pcEmbodiedEntity->GetOriginAnchor());
00119          /* Ground sensor equipped entity */
00120          m_pcGroundSensorEquippedEntity =
00121             new CGroundSensorEquippedEntity(this,
00122                                             "ground_0");
00123          AddComponent(*m_pcGroundSensorEquippedEntity);
00124          m_pcGroundSensorEquippedEntity->AddSensor(CVector2(0.03f, -0.009f),
00125                                                    CGroundSensorEquippedEntity::TYPE_GRAYSCALE,
00126                                                    m_pcEmbodiedEntity->GetOriginAnchor());
00127          m_pcGroundSensorEquippedEntity->AddSensor(CVector2(0.03f,  0.0f),
00128                                                    CGroundSensorEquippedEntity::TYPE_GRAYSCALE,
00129                                                    m_pcEmbodiedEntity->GetOriginAnchor());
00130          m_pcGroundSensorEquippedEntity->AddSensor(CVector2(0.03f,  0.009f),
00131                                                    CGroundSensorEquippedEntity::TYPE_GRAYSCALE,
00132                                                    m_pcEmbodiedEntity->GetOriginAnchor());
00133          /* RAB equipped entity */
00134          m_pcRABEquippedEntity = new CRABEquippedEntity(this,
00135                                                         "rab_0",
00136                                                         un_rab_data_size,
00137                                                         f_rab_range,
00138                                                         m_pcEmbodiedEntity->GetOriginAnchor(),
00139                                                         *m_pcEmbodiedEntity,
00140                                                         CVector3(0.0f, 0.0f, RAB_ELEVATION));
00141          AddComponent(*m_pcRABEquippedEntity);
00142          /* Controllable entity
00143             It must be the last one, for actuators/sensors to link to composing entities correctly */
00144          m_pcControllableEntity = new CControllableEntity(this, "controller_0");
00145          AddComponent(*m_pcControllableEntity);
00146          m_pcControllableEntity->SetController(str_controller_id);
00147          /* Update components */
00148          UpdateComponents();
00149       }
00150       catch(CARGoSException& ex) {
00151          THROW_ARGOSEXCEPTION_NESTED("Failed to initialize entity \"" << GetId() << "\".", ex);
00152       }
00153    }
00154 
00155    /****************************************/
00156    /****************************************/
00157 
00158    void CEPuckEntity::Init(TConfigurationNode& t_tree) {
00159       try {
00160          /*
00161           * Init parent
00162           */
00163          CComposableEntity::Init(t_tree);
00164          /*
00165           * Create and init components
00166           */
00167          /* Embodied entity */
00168          m_pcEmbodiedEntity = new CEmbodiedEntity(this);
00169          AddComponent(*m_pcEmbodiedEntity);
00170          m_pcEmbodiedEntity->Init(GetNode(t_tree, "body"));
00171          /* Wheeled entity and wheel positions (left, right) */
00172          m_pcWheeledEntity = new CWheeledEntity(this, "wheels_0", 2);
00173          AddComponent(*m_pcWheeledEntity);
00174          m_pcWheeledEntity->SetWheel(0, CVector3(0.0f,  HALF_INTERWHEEL_DISTANCE, 0.0f), WHEEL_RADIUS);
00175          m_pcWheeledEntity->SetWheel(1, CVector3(0.0f, -HALF_INTERWHEEL_DISTANCE, 0.0f), WHEEL_RADIUS);
00176          /* LED equipped entity, with LEDs [0-11] and beacon [12] */
00177          m_pcLEDEquippedEntity = new CLEDEquippedEntity(this, "leds_0");
00178          AddComponent(*m_pcLEDEquippedEntity);
00179          m_pcLEDEquippedEntity->AddLEDRing(
00180             CVector3(0.0f, 0.0f, LED_RING_ELEVATION),
00181             LED_RING_RADIUS,
00182             LED_RING_START_ANGLE,
00183             8,
00184             m_pcEmbodiedEntity->GetOriginAnchor());
00185          /* Proximity sensor equipped entity */
00186          m_pcProximitySensorEquippedEntity =
00187             new CProximitySensorEquippedEntity(this,
00188                                                "proximity_0");
00189          AddComponent(*m_pcProximitySensorEquippedEntity);
00190          m_pcProximitySensorEquippedEntity->AddSensorRing(
00191             CVector3(0.0f, 0.0f, PROXIMITY_SENSOR_RING_ELEVATION),
00192             PROXIMITY_SENSOR_RING_RADIUS,
00193             PROXIMITY_SENSOR_RING_START_ANGLE,
00194             PROXIMITY_SENSOR_RING_RANGE,
00195             8,
00196             m_pcEmbodiedEntity->GetOriginAnchor());
00197          /* Light sensor equipped entity */
00198          m_pcLightSensorEquippedEntity =
00199             new CLightSensorEquippedEntity(this,
00200                                            "light_0");
00201          AddComponent(*m_pcLightSensorEquippedEntity);
00202          m_pcLightSensorEquippedEntity->AddSensorRing(
00203             CVector3(0.0f, 0.0f, PROXIMITY_SENSOR_RING_ELEVATION),
00204             PROXIMITY_SENSOR_RING_RADIUS,
00205             PROXIMITY_SENSOR_RING_START_ANGLE,
00206             PROXIMITY_SENSOR_RING_RANGE,
00207             8,
00208             m_pcEmbodiedEntity->GetOriginAnchor());
00209          /* Ground sensor equipped entity */
00210          m_pcGroundSensorEquippedEntity =
00211             new CGroundSensorEquippedEntity(this,
00212                                             "ground_0");
00213          AddComponent(*m_pcGroundSensorEquippedEntity);
00214          m_pcGroundSensorEquippedEntity->AddSensor(CVector2(0.03f, -0.009f),
00215                                                    CGroundSensorEquippedEntity::TYPE_GRAYSCALE,
00216                                                    m_pcEmbodiedEntity->GetOriginAnchor());
00217          m_pcGroundSensorEquippedEntity->AddSensor(CVector2(0.03f,  0.0f),
00218                                                    CGroundSensorEquippedEntity::TYPE_GRAYSCALE,
00219                                                    m_pcEmbodiedEntity->GetOriginAnchor());
00220          m_pcGroundSensorEquippedEntity->AddSensor(CVector2(0.03f,  0.009f),
00221                                                    CGroundSensorEquippedEntity::TYPE_GRAYSCALE,
00222                                                    m_pcEmbodiedEntity->GetOriginAnchor());
00223          /* RAB equipped entity */
00224          Real fRange = 0.8f;
00225          GetNodeAttributeOrDefault(t_tree, "rab_range", fRange, fRange);
00226          UInt32 unDataSize = 2;
00227          GetNodeAttributeOrDefault(t_tree, "rab_data_size", unDataSize, unDataSize);
00228          m_pcRABEquippedEntity = new CRABEquippedEntity(this,
00229                                                         "rab_0",
00230                                                         unDataSize,
00231                                                         fRange,
00232                                                         m_pcEmbodiedEntity->GetOriginAnchor(),
00233                                                         *m_pcEmbodiedEntity,
00234                                                         CVector3(0.0f, 0.0f, RAB_ELEVATION));
00235          AddComponent(*m_pcRABEquippedEntity);
00236          /* Controllable entity
00237             It must be the last one, for actuators/sensors to link to composing entities correctly */
00238          m_pcControllableEntity = new CControllableEntity(this);
00239          AddComponent(*m_pcControllableEntity);
00240          m_pcControllableEntity->Init(GetNode(t_tree, "controller"));
00241          /* Update components */
00242          UpdateComponents();
00243       }
00244       catch(CARGoSException& ex) {
00245          THROW_ARGOSEXCEPTION_NESTED("Failed to initialize entity \"" << GetId() << "\".", ex);
00246       }
00247    }
00248 
00249    /****************************************/
00250    /****************************************/
00251 
00252    void CEPuckEntity::Reset() {
00253       /* Reset all components */
00254       CComposableEntity::Reset();
00255       /* Update components */
00256       UpdateComponents();
00257    }
00258 
00259    /****************************************/
00260    /****************************************/
00261 
00262    void CEPuckEntity::Destroy() {
00263       CComposableEntity::Destroy();
00264    }
00265 
00266    /****************************************/
00267    /****************************************/
00268 
00269 #define UPDATE(COMPONENT) if(COMPONENT->IsEnabled()) COMPONENT->Update();
00270 
00271    void CEPuckEntity::UpdateComponents() {
00272       UPDATE(m_pcRABEquippedEntity);
00273       UPDATE(m_pcLEDEquippedEntity);
00274    }
00275 
00276    /****************************************/
00277    /****************************************/
00278    
00279    REGISTER_ENTITY(CEPuckEntity,
00280                    "e-puck",
00281                    "Carlo Pinciroli [ilpincy@gmail.com]",
00282                    "1.0",
00283                    "The e-puck robot.",
00284                    "The e-puck is a open-hardware, extensible robot intended for education. In its\n"
00285                    "simplest form, it is a two-wheeled robot equipped with proximity sensors,\n"
00286                    "ground sensors, light sensors, a microphone, a frontal camera, and a ring of\n"
00287                    "red LEDs. More information is available at http://www.epuck.org\n\n"
00288                    "REQUIRED XML CONFIGURATION\n\n"
00289                    "  <arena ...>\n"
00290                    "    ...\n"
00291                    "    <e-puck id=\"eb0\">\n"
00292                    "      <body position=\"0.4,2.3,0.25\" orientation=\"45,90,0\" />\n"
00293                    "      <controller config=\"mycntrl\" />\n"
00294                    "    </e-puck>\n"
00295                    "    ...\n"
00296                    "  </arena>\n\n"
00297                    "The 'id' attribute is necessary and must be unique among the entities. If two\n"
00298                    "entities share the same id, initialization aborts.\n"
00299                    "The 'body/position' attribute specifies the position of the pucktom point of the\n"
00300                    "e-puck in the arena. When the robot is untranslated and unrotated, the\n"
00301                    "pucktom point is in the origin and it is defined as the middle point between\n"
00302                    "the two wheels on the XY plane and the lowest point of the robot on the Z\n"
00303                    "axis, that is the point where the wheels touch the floor. The attribute values\n"
00304                    "are in the X,Y,Z order.\n"
00305                    "The 'body/orientation' attribute specifies the orientation of the e-puck. All\n"
00306                    "rotations are performed with respect to the pucktom point. The order of the\n"
00307                    "angles is Z,Y,X, which means that the first number corresponds to the rotation\n"
00308                    "around the Z axis, the second around Y and the last around X. This reflects\n"
00309                    "the internal convention used in ARGoS, in which rotations are performed in\n"
00310                    "that order. Angles are expressed in degrees. When the robot is unrotated, it\n"
00311                    "is oriented along the X axis.\n"
00312                    "The 'controller/config' attribute is used to assign a controller to the\n"
00313                    "e-puck. The value of the attribute must be set to the id of a previously\n"
00314                    "defined controller. Controllers are defined in the <controllers> XML subtree.\n\n"
00315                    "OPTIONAL XML CONFIGURATION\n\n"
00316                    "You can set the emission range of the range-and-bearing system. By default, a\n"
00317                    "message sent by an e-puck can be received up to 80cm. By using the 'rab_range'\n"
00318                    "attribute, you can change it to, i.e., 4m as follows:\n\n"
00319                    "  <arena ...>\n"
00320                    "    ...\n"
00321                    "    <e-puck id=\"eb0\" rab_range=\"4\">\n"
00322                    "      <body position=\"0.4,2.3,0.25\" orientation=\"45,90,0\" />\n"
00323                    "      <controller config=\"mycntrl\" />\n"
00324                    "    </e-puck>\n"
00325                    "    ...\n"
00326                    "  </arena>\n\n"
00327                    "You can also set the data sent at each time step through the range-and-bearing"
00328                    "system. By default, a message sent by an e-puck is 2 bytes long. By using the"
00329                    "'rab_data_size' attribute, you can change it to, i.e., 20 bytes as follows:\n\n"
00330                    "  <arena ...>\n"
00331                    "    ...\n"
00332                    "    <e-puck id=\"eb0\" rab_data_size=\"20\">\n"
00333                    "      <body position=\"0.4,2.3,0.25\" orientation=\"45,90,0\" />\n"
00334                    "      <controller config=\"mycntrl\" />\n"
00335                    "    </e-puck>\n"
00336                    "    ...\n"
00337                    "  </arena>\n\n",
00338                    "Under development"
00339       );
00340 
00341    /****************************************/
00342    /****************************************/
00343 
00344    REGISTER_STANDARD_SPACE_OPERATIONS_ON_COMPOSABLE(CEPuckEntity);
00345 
00346    /****************************************/
00347    /****************************************/
00348 
00349 }
```

---

Generated on 10 Jul 2018 for ARGoS by 
 1.6.1
